# Electronic clinical outcome assessments and the European Union's clinical trial regulation: Smoothing the path to friction?

**DOI:** 10.1016/j.conctc.2026.101623

**Published:** 2026-02-28

**Authors:** Pierre Mermet-Bouvier, Pierre-Frederic Omnes, Valdo Arnera, Scottie Kern

**Affiliations:** a ICON PLC, Dublin, Ireland; b TransPerfect Life Sciences, Paris, France; c Clario, Geneva, Switzerland; d Critical Path Institute, Tucson, AZ, USA

**Keywords:** Electronic clinical outcome assessments, eCOA, European Union, Clinical trials regulation, Clinical trial information system, Ethics committees, Clinical trial application

## Abstract

The European Union's Clinical Trial Regulation 536/2014 (EU-CTR) sought to harmonize clinical trial rules, ensure high standards of safety, and streamline processes through its new Clinical Trials Information System (CTIS). However, confusion remains regarding submission requirements for “patient-facing documents” in Parts I and II of clinical trial applications (CTAs). Misinterpretations—particularly around what qualifies as written information—have led to unnecessary submissions of materials like eCOA or ePRO “screenshots,” which are not legally required.

The EU's EUDRALEX Volume 10 Q&A (notably Question 1.24) clarified that only patient-facing documents linked to trial endpoints, such as questionnaires, diaries, or patient cards, need inclusion in Part I alongside the protocol. There is no legal basis to demand all patient materials or their translations in Part II. Despite this clarification, member states retain autonomy under Article 26 to set national translation and document requirements, leading to inconsistent practices and continued administrative burden.

Recent initiatives, including CTR Collaborate (under the Accelerating Clinical Trials in the EU program) and MedEthics EU, seek to promote harmonization and efficient assessment. Empirical studies show that Ethics Committees (ECs) generally do not expect ePRO screenshots—most require only translated questionnaires.

EU-CTR's success depends on collective awareness and streamlined processes. With ICH E6 Revision 3 and global efforts by organizations like the WHO, there is an opportunity to balance regulatory rigor with practical efficiency—reducing redundant submissions and emphasizing participant protection as the core ethical priority.

## 1 Introduction

The European Union (EU) introduced the Clinical Trial Regulation 536/2014 (EU-CTR) [1] to replace the legacy Clinical Trial Directive 2001/20/EC which had governed the execution of clinical trials within the EU since 2004. The EU-CTR aims to further harmonize the rules for conduct of clinical trials in the EU, as well as maintaining high standards of quality and safety for medicinal product development. Additionally, EU-CTR aims to reduce the administrative burden around clinical trial initiation and execution by streamlining regulatory processes and workflows via a new centralized portal and database called the Clinical Trials Information System (CTIS). The end of the transition period for EU-CTR – whereby all active trials were to be transitioned to EU-CTR – was January 30, 2025. Here, the Critical Path Institute's Electronic Clinical Outcome Assessment (eCOA) Consortium investigates some of the unintended consequences of CTIS implementation.

## 2 The problem

Despite the ambitions of the EU-CTR and CTIS, confusion persists around what actually needs to be submitted in Parts 1 or 2 for a clinical trial in relation to “patient-facing documents”. Alignment on what this term actually refers to has been a persistent challenge within the eCOA space in a global context for some time. A 2020 article [2] co-authored by eCOA Consortium colleagues examined the misinterpretation of the term “written information” found within ICH E6 R2, concluding that a broad misinterpretation led Institutional Review Boards (IRBs) and Ethics Committees (ECs) to require submission of *any* written material presented to a trial participant, when the requirement actually relates to consent and trial recruitment only, not clinical data capture. Consequently, the unfounded mindset that clinical trial submissions must incorporate final versions of all types of translated patient documents, including “screenshots” (visual representations of eCOA screens that trial participants would see) persists. The hope was that EU-CTR would provide more harmonized requirements, but the reverse has become true - EU-CTR remains loosely interpreted with significant uncertainties as to what to include in clinical trial applications (CTAs), such as other types of patient documents not listed in EU CTR Annex I or EU CTR Q&A 1.24 (i.e., instructions to complete questionnaires, screenshots for electronic versions of ePRO/eCOA, etc.) but nonetheless requested via ECs guidance or Requests for Information in some member states.

## 3 Impact of CTR on eCOA

Resources are available to sponsors to support successful compilation of CTAs. Of note is the EUDRALEX-Volume 10 EU-CTR Questions & Answers [3] document (version 6.9) born from discussions within the Clinical Trials Expert Group (CTEG), a group chaired by the European Commission (EC) and composed of representatives of all EU Member States, European Economic Area contracting parties and EMA.

**Fig. 1:**
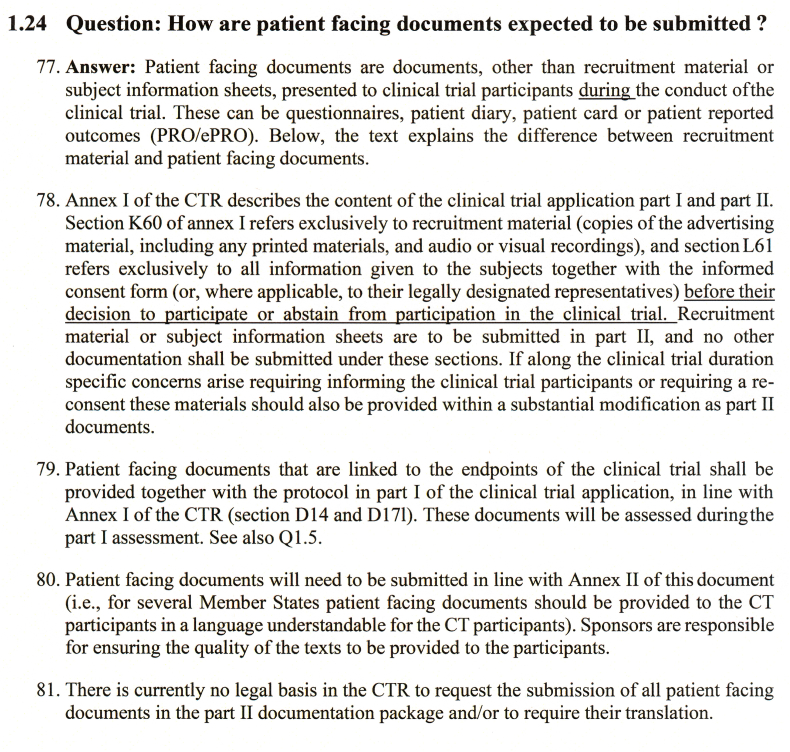
Extracted question 1.24 from *Clinical Trials Regulation (EU) No 536/2014 Questions and Answers Version 7.1*.

The issue of disharmonized patient document requirements has been raised repeatedly by sponsors’ stakeholders to the EC and CTEG, which led to a CTEG consensus position documented in Question 1.24 of that Q&A. This position goes beyond the original requirements for patient documents described in EU-CTR Annex I item L, per Fig. 1.

This interpretation, absent from EU-CTR Annex I and introduced in Line 79 of the Q&A, derives from clinical trial protocol considerations within Annex I D (i.e., not specifically mandating the provision of additional documents). Line 79 states: “*Patient facing documents that are linked to the endpoints of the clinical trial shall be provided together with the protocol in part 1 of the clinical trial application.”* Crucially, line 81 verifies that there is “no legal basis in the CTR to request submission of all patient-facing documents in the Part II documentation package and/or to require their translation”.

While the EU Regulation focuses on patient documents relevant to consent and recruitment, the interpretation detailed in Q1.24 extends beyond the legally binding text to align different EU member state viewpoints on the matter. The statements in lines 79 and 81 comprehensively defeat the misguided assumption that documents like final, translated “screenshots” are to be submitted in Part II. The Q&A documents themselves are not legally binding, affording latitude to national health entities to continue to issue country-specific requirements for patient documents either in the CTA Part I – core dossier (protocol endpoints) or Part II – country-specific documents (consent and recruitment). The EUDRALEX Volume 10 Q&A referred to above is a fluid document and susceptible to future changes.

National-level expectations on endpoint-related patient documents.

**Fig. 2:**
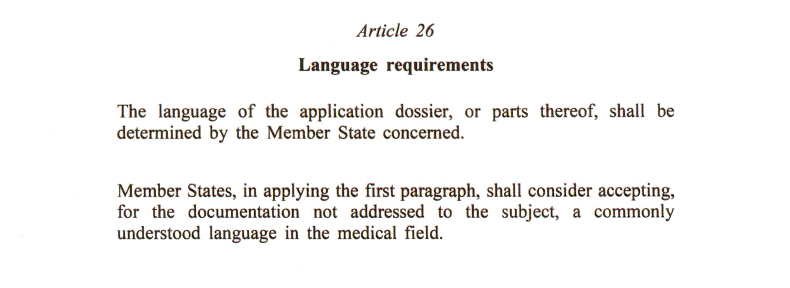
Article 26 of Regulation (EU) No 536/2014 on clinical trials on medicinal products for human use.

Article 26 of the EU-CTR offers latitude to Member States regarding translation requirements of mandated documents to be included in the CTA (EU-CTR Annex I for initial submissions, and in EU-CTR Annex II for substantial modifications) as detailed in Fig. 2.

The endpoint-related patient-facing documents described in EUDRALEX Volume 10 Q&A Q1.24 are limited to questionnaires, patient diaries, patient cards or patient-reported outcomes. This requirement, as detailed in Q&A 1.24, is an interpretation of EU CTR Annex I introduced due to multiple questions arising from applicants after EU CTR went live, and has created additional requirements not foreseen in EU CTR Annex I. Reinterpretation or at least clarification of the current Q&A 1.24 would avoid persistent questions in submission package preparation. A full list of applicable documents may be the ultimate solution.

Annex II of the Q&A (Language requirements for Part I documents), details translation requirements for core dossier documents (Part I), such as the clinical trial protocol, but also patient facing documents relating to endpoints. The extension of patient document requirements beyond consent and recruitment (as per EUDRALEX Q&A Q1.24) triggers its own set of translation requirements per country. Document translations therefore remain on the critical path for submission readiness for those endpoint-related documents, impacting Part I completion based on certain country requirements. Interestingly, the proposal for the European Biotech Act released in December 2025 suggests moving all translated documents into Part II, which some sponsors did during the first months of the CTIS rollout and before the Q&A was updated with Q1.24 in September 2022. When the European Biotech Act is finalized, the EUDRALEX Volume 10 Q&A will be largely updated, including Question 1.24.

Additionally, translation requirements as described in Q&A Annex 2 include differing options like English only, English *and* national language, English *or* national language, or national language only, and this does not factor in any country-specific or even EC-specific requirements (some countries have multiple ECs available for selection to review a CTA). Consequently, some degree of disharmony persists between countries or even between ECs within a single country. We are not yet at steady state, and stability is necessary to allow for alignment.

To compile the CTIS application, the following principles and questions may apply.

• It is important to define upfront for the clinical trial which patient documents fall under the “endpoint-related” category to determine their inclusion in the application, and their translation to support the planned submission date
• Endpoint-related patient-facing documents (both English and translated versions) must be included in the same section as the protocol in CTIS (document type “protocol”). Consequently, those documents fall under CTIS transparency rules [4] meaning they will eventually be made public (as early as the trial decision from the first country for phase 2-4 trials). Specific attention to redaction of those documents may apply in case of commercially confidential information or protected personal data [5] being exposed.

Since September 20, 2024, additional features were implemented on the CTIS public portal, notably the availability of advanced searches (e.g., specific searches for “eCOA” or “ePRO”). Other potentially patient facing documents not listed in Q1.24 and sometimes not even linked to endpoints (e.g., drug administration, procedure-specific guides for patients, etc.) may be considered as country-specific requirements through the assessment of a CTA - those documents may be required in the protocol section, as there is currently no other placeholder for this information in other sections of the clinical trial application within CTIS.

The continuing disharmony across Member States in relation to EU-CTR implementation, CTA requirements and assessment practices (including translations) has been widely acknowledged and documented by all stakeholders. Initiatives exist at the EU level to holistically address CTR implementation issues.

• CTR Collaborate [6], created by the Clinical Trials Coordination Group [7] and anchored under the “Accelerating Clinical Trials in the EU” (ACT EU) [8] initiative, aims to develop effective assessment and supervision procedures for clinical trials, with close collaboration between and within Member States involving national competent authorities and ECs. Sponsors are also represented.
• MedEthics EU [9], a group of national representatives of medical research ECs that convene for discussion and mutual learning between EU/EEA Member States ECs to drive harmonization in the ethics review.

A recent CTR Collaborate Stakeholder meeting [10], supported by the ACT EU, incorporated an in-depth analysis of the opportunities for procedural improvement and greater harmonization, and as such provides regulator-acknowledged evidence of what needs to change.

### 3.1 European EC expectations

From an EC perspective, we have empirical evidence of the lack of expectations for ePRO screenshot submission, via a study run in 2023 that aimed to identify and collect information from selected ECs on requirements for ePRO screenshot submission [11]. Five ECs were randomly selected from each of 5 countries (UK, Spain, Netherlands, Germany and Poland). Stage 1 of the study reviewed the submissions checklists for the 25 ECs, and not one had any reference to or requirements for ePRO screenshots. In stage 2, all 25 ECs were contacted to confirm the currency of their checklist; 20 ECs responded and all of them confirmed their checklist were still current. Stage 3 saw the 20 responding ECs asked if a new clinical trial that incorporated ePROs arose, was there anything they would request that was not in the checklist, with 16 responding and 6 (6/20) ECs confirming that no ePRO-related information was expected. Several ambiguous answers were received (10/20), so a clarifying closed question asked what detail they would request specific to ePROs – of those that responded (9/10), all those ECs indicated that “a PDF version of the *paper* questionnaires” was sufficient. In conclusion, not one of the ECs engaged (16/20) specifically indicated that screenshots of the ePROs were expected.

### 3.2 A pathway forward

Getting better medical treatments to patients faster is the common goal. There is a collective desire to see avoidable inefficiencies and redundant processes eradicated, and EU-CTR is a clear attempt to achieve that. Member State-level autonomy is a fundamental cornerstone of the EU, but we must work collectively to generate awareness of avoidable delays to clinical trial execution. As an industry, we must identify and implement new approaches that respect legal requirements but get us to the end goal quickly. As detailed above, some EU-level initiatives will reap benefits in time, but we must shift the mindset.

Revision 3 of ICH E6 came into effect in EU on July 23, 2025 and in Switzerland on August 15, 2025 and provides an opportunity to further front-load quality via the qualification and assessment of service providers [12]. On an EC level, this may be applied to translations, but we should ask what the ECs’ role really is in verifying translations; the best practice of linguistic validation of valid PRO measures absorbs the risk, and the burden of proof surely sits with sponsors selecting competent translation service providers.

Active EU-level initiatives seek to collate and react to the challenges addressed, but an unclear environment will persist until practical outcomes come from these initiatives surface. There are also global efforts, such as the World Health Organization's work to develop best practices for clinical trials [13], where review of the pertinent content (2.2 Good clinical trials respect the rights and well-being of participants) logically focuses on participant communication, consent and safety, and not documents that support trial endpoints.

There is a balance to be struck between the need for regulation to evolve and accommodate improvements to process that increase that would support enhances to efficient clinical trial startup, and the need for stability, principally in the form of even greater harmonization.

Our intention with this article is to highlight where simple changes of mindset and understanding will, in the European context, reduce the collective administrative burden of trial start up while honoring the true role of an EC – protecting the trial participant.

## CRediT authorship contribution statement

**Pierre Mermet-Bouvier:** Writing – review & editing, Writing – original draft, Conceptualization. **Pierre-Frederic Omnes:** Writing – review & editing, Writing – original draft, Conceptualization. **Valdo Arnera:** Writing – review & editing, Conceptualization. **Scottie Kern:** Writing – review & editing, Writing – original draft, Supervision, Conceptualization.

## Funding statement

This research received no specific grant from any funding agency in the public or commercial sectors. The eCOA Consortium is solely funded by the membership fees paid by its members.

## Declaration of competing interest

The authors declare that they have no known competing financial interests or personal relationships that could have appeared to influence the work reported in this paper.

## Data Availability

No data was used for the research described in the article.
